# Interventions and assessment tools addressing key concepts people need to know to appraise claims about treatment effects: a systematic mapping review

**DOI:** 10.1186/s13643-016-0389-z

**Published:** 2016-12-29

**Authors:** Astrid Austvoll-Dahlgren, Allen Nsangi, Daniel Semakula

**Affiliations:** 1 Knowledge Centre for the Health Services, Norwegian Institute of Public Health, BOKS 7004 St. Olavsplass, 0130 Oslo, Norway; 2 Makerere University College of Health Sciences, New Mulago Hospital Complex, P.O. Box 7072, Kampala, Uganda

**Keywords:** Health literacy, Evidence-based medicine, Controlled trials, Patient education, Decision making

## Abstract

**Background:**

People’s ability to appraise claims about treatment effects is crucial for informed decision-making. Our objective was to systematically map this area of research in order to (a) provide an overview of interventions targeting key concepts that people need to understand to assess treatment claims and (b) to identify assessment tools used to evaluate people’s understanding of these concepts. The findings of this review provide a starting point for decisions about which key concepts to address when developing new interventions, and which assessment tools should be considered.

**Methods:**

We conducted a systematic mapping review of interventions and assessment tools addressing key concepts important for people to be able to assess treatment claims. A systematic literature search was done by a reserach librarian in relevant databases. Judgement about inclusion of studies and data collection was done by at least two researchers. We included all quantitative study designs targeting one or more of the key concepts, and targeting patients, healthy members of the public, and health professionals. The studies were divided into four categories: risk communication and decision aids, evidence-based medicine and critical appraisal, understanding of controlled trials, and science education. Findings were summarised descriptively.

**Results:**

We included 415 studies, of which the interventions and assessment tools we identified included only a handful of the key concepts. The most common key concepts in interventions were “Treatments usually have beneficial and harmful effects,” “Treatment comparisons should be fair,” “Compare like with like,” and “Single studies can be misleading.” A variety of assessment tools were identified, but only four assessment tools included 10 or more key concepts.

**Conclusions:**

There is great potential for developing learning and assessment tools targeting key concepts that people need to understand to assess claims about treatment effects. There is currently no instrument covering assessment of all these key concepts.

**Electronic supplementary material:**

The online version of this article (doi:10.1186/s13643-016-0389-z) contains supplementary material, which is available to authorized users.

## Background

A large number of studies conducted across different populations and contexts have concluded that people’s ability to assess and apply health information is generally poor [1–10]. This is particularly the case when it comes to key concepts related to understanding the effects of treatments, such as understanding the need for fair comparisons of treatments, judging whether a comparison of treatments is a fair comparison, and understanding the role of chance, and the results and relevance of fair comparisons of treatments [1–10]. Furthermore, many people rely on anecdotes, as opposed to information based on research, and may overrate the trustworthiness of the information they find [3, 11–14]. As a result, people may be poorly informed and may trust information that is incomplete or even harmful. For example, studies have found that people may not have insight into reasons for policy switches between using brand or generic drugs, or the efficacy of preventive treatments such as screening interventions or vaccination [10, 14–16]. Furthermore, people’s lack of understanding of research methods, such as randomisation, may also be a barrier to people’s participation in controlled trials addressing treatment uncertainties [7].

Knowing what to trust and being able to assess if a claim is based on a review of fair comparisons of treatments is the first step in making an informed decision [17, 18]. Studies have found that patients may play an important part in promoting evidence-based practice important for patient safety but also for quality of care [19–21]. Studies also suggest that patients who are more informed are more involved, experience less decisional conflict, and choose less invasive treatments [16]. However, decisions about healthcare do not only happen on the individual level, many patients today have great influence on system level decisions, for example, through demand of new services and treatments, as participants in priority setting of research, members of hospital boards and as communicators of health information to fellow patients [22]. Considering that many patients do not rely on the best available evidence when making these decisions, the consequences may be costly if people are left uninformed.

Research exploring peoples’ ability to assess treatment effects is challenged by partly overlapping and sometimes parallel research areas being responsible for studies that have often focused on a specific concept, such as understanding of risk or randomization [7, 23–27]. Moreover, until recently, no consensus or conceptualisation of the key concepts critical to understanding the effects of treatments has been available [28]. Given that this research is characterized by heterogeneity, and considering the need for interventions and appropriate assessment tools in this area, we set out to conduct a systematic mapping review of interventions and assessment tools used in such studies [29]. The framework for this review, guiding the identification of interventions and assessment tools was based on a previously published list, or syllabus, we created of the key concepts we believe is important for people to be able to understand to assess treatment claims [30]. This work was done as part of the Informed Healthcare Choices (IHC) project. The IHC project aims to support the use of research evidence by patients and the public, policymakers, journalists and health professionals. The multidisciplinary group responsible for the project includes researchers in six countries—Norway, Uganda, Kenya, Rwanda, UK and Australia. The project has been responsible for developing and evaluating educational resources to improve the ability of people in low-income countries to assess claims about treatment effects (Semakula D, Nsangi A, Oxman M, Austvoll-Dahlgren A, Rosenbaum S, Kaseje M, et al.: Can an educational podcast improve the ability of parents of primary school children to assess claims about the benefits and harms of treatments?, submitted), (Nsangi A, Semakula D, Oxman M, Austvoll-Dahlgren A, Rosenbaum S, Kaseje M, et al.: Evaluation of resources to teach children in low income countries to assess claims about treatment effects, submitted). A short list of the concepts is presented in an additional word file (see Additional file 1). We defined treatment as “any action intended to improve health.”

### Objective

Our objective was to systematically map this area of research by applying the list of key concepts, in order to (a) provide an overview of interventions targeting key concepts that people need to understand to assess treatment claims and (b) to identify assessment tools used to evaluate people’s understanding of these concepts.

## Methods

There is an increasing variety of types of reviews for different purposes [29, 31]. If systematic, reviews should not only inform decisions about healthcare but also serve as the starting point when initiating new research such as developing interventions or assessment tools [18, 29, 32]. In order to address the above mentioned objectives, we set out to perform a mapping review. According to the typology of reviews by Grant and Booth [29], a mapping review differs from other scoping reviews in that the subsequent outcome of the review may require more work such as identifying gaps in the research literature or further review work. There is no agreement on standard method of doing a mapping review, as this will depend on the objectives. However, generally, mapping reviews provides an overview of the literature and identify gaps. Such reviews may also describe and organise the literature according to theoretical perspectives, population or other characteristics [29].

### Review design, search strategy, and inclusion criteria

**Fig. 1:**
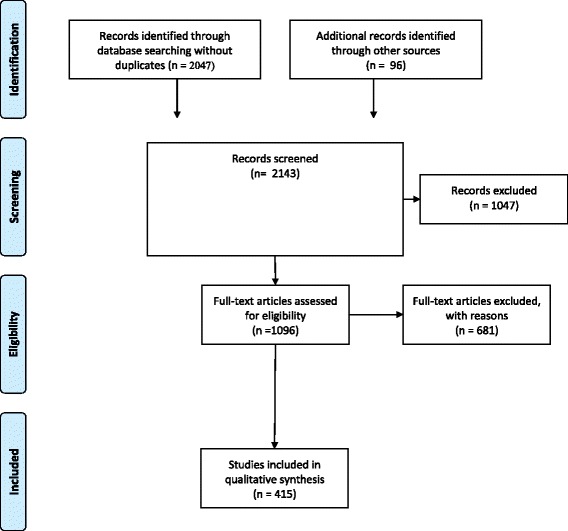
PRISMA flow diagram

The protocol for this review was registered in PROSPERO [33]. The PRISMA flow diagram can be found in Fig. 1.

#### Search strategy and inclusion criteria

We conducted a focused search for interventions and assessment tools targeting one or more of the key concepts. The full search strategy can be seen in more detail in an additional word file (see Additional file 2). A research librarian developed a search strategy based on the overview of key concepts. CDSR, DARE, HTA, CENTRAL, Method studies (Cochrane Library), MEDLINE 1946 to 22.06.13 (Ovid), and ERIC were included in our search. We applied the observational filter from SIGN, and the Cochrane filter based on HSSS (as applied to MEDLINE). For ERIC, we adapted Cochrane HSSS and SIGN from Medline to ERIC.

In order to identify unpublished studies, we also contacted key researchers working in related research areas such as health literacy and training of patients and consumers in evidence-based medicine, including members of the Cochrane Consumer group and the Nordic Health Literacy Network. We also checked the reference lists of all relevant systematic reviews. Hand searches were finalized in September 2015. The list of key concepts was revised after this review had been completed (revisions included adding concept 1.9, and splitting concept 5.1 into concepts 1.1 and 5.1—see Additional file 2 for more detail). As a result, the new key concept was not included in our search and data collection, and concepts 1.1 and 5.1 were treated as a single key concept. This did not influence our search strategy or conclusions as the revisions of the Key Concepts’ list was done after we had completed the review.

#### Types of designs

We included all quantitative study designs, including controlled trials and observational studies. We included both studies evaluating an intervention and descriptive studies without any intervention but which assessed understanding of one or more of the key concepts in a population. We also included studies describing the development of assessment tools.

#### Types of participants

Studies of patients and healthy members of the public were included. We also included studies aimed at health professionals, since the interventions and assessment tools directed at them may also be applicable to a lay public.

#### Types of interventions

We included all interventions that included one or more key concepts.

#### Types of assessment tools

We included all assessment tools that evaluated peoples’ understanding of one or more of the key concepts. We conceptualised “understanding” as any measure that assessed people’s knowledge or ability to apply the key concepts.

#### Exclusion criteria

We excluded theoretical or conceptual papers, editorials, letters, and studies with qualitative designs. We also excluded interventions that did not directly address any of the key concepts, for example framing interventions or others that intended to persuade and not educate people about making informed choices. For pragmatic reasons, we did not include publications in languages other than English or the Scandinavian languages.

### Data collection and presentation of findings

All references were reviewed independently by two researchers (AA and AN). Studies classified as clearly relevant or unclear were retrieved in full text. At least two researchers (AA, AN, DS) screened and considered for inclusion all publications retrieved in full text. Any difference in opinion between two researchers was discussed with a third.

Data collection was performed by one researcher (AA, AN, or DS) using a data collection form, extracting information on the purpose of the study (intervention study or descriptive study), study design, population (patient, professionals or others), intervention, outcomes, and assessment tools. All data collection forms were double-checked by another researcher.

We categorized the interventions using pragmatic criteria and predefined categories informed by our knowledge of the research area using the following categories:

- Risk communication and decision aids (risk and DA): studies evaluating the effects of interventions facilitating informed choice (mostly targeting patients) including how best to present estimates of risk and use of decision aids. Although the content and purpose of such interventions varies, they usually explore different ways of presenting the effects of treatments, help patients clarify their values and preferences, and provide a structured path through the decision making process.
- Understanding of trials: studies evaluating interventions to improve people’s understanding of trial methodology and informed consent. The research interests underlying many of these studies are to improve recruitment to randomized trials and to identify barriers to consent. One reason for this is that one of most important reasons that people reject participating in trials is poor understanding of trial methods, and the benefits and harms associated with participation [7].
- Evidence-based medicine and critical appraisal (EBM and CA): studies evaluating interventions that typically aim to enable people (usually health professionals) to formulate clinical questions, search for relevant evidence, appraise, and apply this evidence in practice [34].
- Science education: studies evaluating interventions that aim to facilitate reasoning or critical thinking, usually in school settings. Such education may take place as part of the existing curricula or be initiated by external research initiatives.

The common goal of these research fields is that they aim to support people in making informed decisions by developing and evaluating interventions that enable people to appraise and apply research evidence [7, 27, 34–36]. These categories were not considered fixed, but were subject to revision if the included studies did not fit well within the categories.

The assignment of interventions to categories was done as part of the data collection process. In most cases, the interventions were categorised according to the purpose of the intervention as stated by the study authors. These categories were not mutually exclusive, and in some cases, an intervention was eligible for more than one category. In cases where an intervention’s assignment was unclear, this was resolved through discussion and consensus among the reviewers.

We also identified outcomes measured in the intervention studies and grouped them according to the following categories: behaviour, attitudes and beliefs, knowledge and skills, costs and other use of resources, and health outcomes.

All interventions and all assessment tools (in intervention studies or descriptive studies) intended to measure people’s understanding of one or more key concepts were tagged by relevant key concepts as part of the data collection process.

The lead researcher and a research assistant (AA, KO) conducted the data entering and summarised the findings using Excel.

## Results

### Description of the included studies

The search strategy resulted in 2143 references of which we judged 1096 potentially relevant and assessed these in full text (see the PRISMA flow diagram in Fig. 1). Of the studies assessed in full-text, we judged 415 to meet the inclusion criteria. The complete list of included studies is shown in an additional excel file (see Additional file 3). Forty-eight were descriptive studies and 367 were intervention studies, targeting one or more of the concepts. Overall, one hundred and twenty-three studies targeted health professionals, 20 studies targeted a mixed group of people including policy makers, and 272 targeted patients or consumers.

### Overview of studies targeting key concepts by intervention categories

**Fig. 2:**
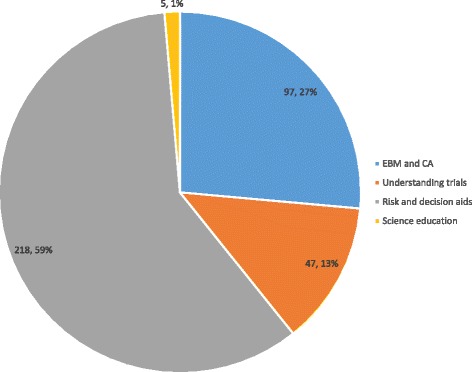
Interventions by category addressing one or more key concepts

Applying the key concept list to the identified research literature, we sorted the studies into four research areas: risk and DA, EBM and CA, understanding of trials, and science education. Although these categories were predefined, the studies we identified fit well, and no new categories emerged as part of the data collection process (see Fig. 2).

### Overview of key concepts in interventions

**Fig. 3:**
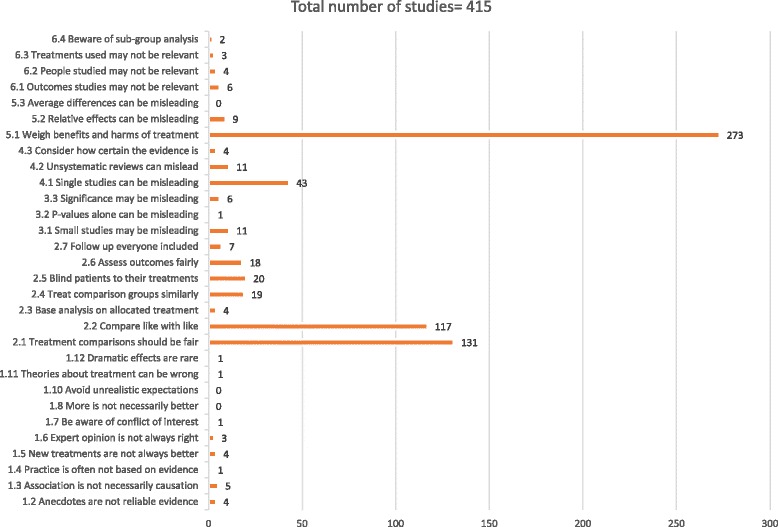
Key concepts in interventions

Overall, the most common key concepts targeted in interventions were concepts 5.1 “Treatments usually have beneficial and harmful effects” (273 studies), concepts 2.1 “Treatment comparisons should be fair” (131 studies), 2.2 “Compare like with like” (117 studies) and concept 4.1 “Single studies can be misleading” (43 studies) (see Fig. 3).

Nearly all key concepts were included in at least one intervention, with the exception of concept 1.8 “More is not necessarily better”, concept 1.10 “Avoid unrealistic expectations”, and concept 5.3 “Average differences can be misleading”.

However, each intervention only targeted a handful of the concepts (ranging from 1 to 14 concepts) (see Additional file 3). Four interventions covered more than 10 key concepts, of which three intended to support people to making informed choices through communication of the results and the certainty of the evidence in decision support tools (three studies) and one was a EBM and CA intervention [37–40].

#### Key concepts in interventions by categories

We included 218 studies of risk and DA interventions. Although this category of studies was the largest, only a few of the key concepts were included and interventions usually focused on one of the key concepts, concept 5.1 “Treatments usually have beneficial and harmful effects.”

The intervention category that included most key concepts was EBM and CA studies. We included 97 such studies. EBM and CA interventions typically included concepts related to judging whether a comparison of treatments is a fair comparison (concepts 2.1 “Treatment comparisons should be fair,” 2.2 “Compare like with like,” 2.4 “Treat comparison groups similarly,” 2.5 “Blind participants to their treatments,” and 2.6 “Assess outcome measures fairly”), concept 4.1 “Single studies can be misleading,” and concept 5.1 “Weigh benefits and harms of treatment].” The majority of these studies targeted health professionals, but some also targeted patients [41–43].

We included 47 studies exploring peoples’ understanding of trials. These interventions targeted mainly three concepts associated with specific areas of consent information, including concepts 2.1 “Treatment comparisons should be fair,” 2.2 “Compare like with like,” and concept 5.1 “Weigh benefits and harms of treatment.”

Five studies were categorised as science education studies and were conducted in school settings. All of them included patients or consumers (students), and four were targeted at younger students (grade 7 and above). The purpose of these interventions was to facilitate critical thinking and science literacy. This may be why this category included most key concepts related to recognizing the need for fair comparisons, including concepts concept 1.2 “Anecdotes are not reliable evidence,” 1.3 “Association is not necessarily causation,” concept 1.6 “Expert opinion is not always right,” and concept 1.7 “Be aware of conflicts of interest,” as well as concepts 2.1 “Treatment comparisons should be fair,” 2.2 “Compare like with like,” and concept 5.1 “Weigh benefits and harms of treatment].”

#### Outcomes evaluated in interventions

**Fig. 4:**
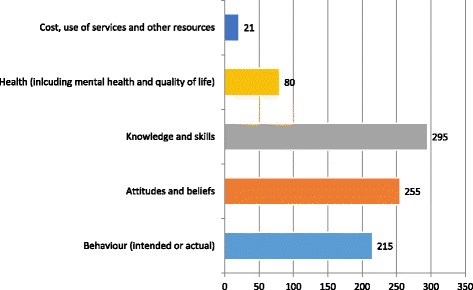
Outcomes and covariates in intervention studies

Factors associated with the interventions and intervention effects were measured on a range of covariates and outcomes. The most common outcomes measured were knowledge and skills (295 studies) and attitudes and beliefs (255 studies), followed by behavioural outcomes (215 studies), health related outcomes including mental health and quality of life (80 studies), and costs, use of services and other resources (21 studies) (see Fig. 4).

### Overview of key concepts in assessment tools

Overall, the number of key concepts included in assessment tools ranged from 0 to 15 concepts. Although some studies included key concepts in their interventions, these were not always included in any of the assessment tools used in the same study. Only four assessment tools included 10 or more key concepts [38, 39, 44, 45]. Two were used for assessing EBM and CA skills and two for assessing understanding of the results and the certainty of the evidence from systematic reviews. An overview of the assessment tools addressing greater than 10 key concepts are presented in Table 1. None of the assessment tools that included 10 or more concepts targeted patients or consumers.

**Table 1:** Assessment tools addressing 10 key concepts or more

Study id (author and year)	Participants	Instrument with concept	Concepts included in measurement	Number of concepts
Ramos 2003, Tilson 2010 (modified version, relative to physical therapists), Dinkevich 2006 (modified version- excluded diagnosis and prevention), Hatmi 2010, Nicholson 2007, Mascola 2008 (adaption including only 7 questions), McCluskey 2005 (did not include advanced statistical questions), Shuval 2010 (adaption), Ilic 2012 (this study only evaluated the two first steps (question formulation and search) [44] (see Additional file 3)	Health professionals	Competency in EBM: the Fresno tool developed as part of the study, using clinical scenarios and open-ended questions related to the five EBM steps. The Fresno test require the candidate to formulate a focused question, identify the most appropriate research design for answering the question, show knowledge of electronic database searching, identify issues important for determining the relevance and validity of a given research article, and discuss the magnitude and importance of research findings. These questions are scored by using a standardised grading system. A series of calculations and fill in the blank questions.	2.1, 2,2, 2.4-2.7, 3.1-3.3, 4.1, 4.2, 5.1, 6.1-6.3	15
Godwin 2003 [45]	Health professionals	Knowledge about EBM: questionnaire with six open ended questions about criteria for quality assessing randomised trials, systematic reviews and diagnostic tests. For each question the respondent would get 0 to 2 points, and six questions evaluating understanding of results scored as 0 or 1 point per question. Instrument probably designed as part of study.	2.1, 2,2, 2.4-2.7, 3.1, 4.1, 4.2, 5.1, 6.1-6.3	13
Rosenbaum 2010 [39]	Mixed	Understanding the findings of trials and systematic reviews: 2 questions about self-assessed understanding of randomised trials rated on a scale, and 7 questions about self-assessed understanding of systematic reviews- including understanding the results, identifying important outcomes, helpfulness and accessibility of the intervention. Correct understanding: four questions about the reviews content- including risk, confidence in the evidence and identification of the most important outcomes. Evaluation tools developed for the study, not further described. Probably used likert scales.	2.1-2.7, 3.1, 4.1- 4.3	11
Vandvik 2012 [38]	Mixed	Understanding of summary of findings (results of systematic reviews): instrument created for study including 6 multiple- choice questions about the certainty of the evidence and interpretation of results, scored as correct/incorrect.	2.1-2.7, 3.1, 4.1- 4.3	11

Assessment tools used in studies targeting patients or consumers included only seven or fewer key concepts. The large majority of these were risk and DA studies, and generally only touching upon one concept 5.1 “Weigh benefits and harms of treatment,” such as the Decisional Conflict Scale [46].

With few exceptions, people’s understanding of the key concepts was evaluated by different measurement instruments and procedures across studies. Many of these were developed for the specific study, and we identified approximately 210 discrete instruments/procedures. The most frequently used assessment tools, were the Fresno tool, which measures competency in EBM (used in nine studies), and the Decisional Conflict Scale, which measures the amount of uncertainty a person has regarding a course of action and the factors contributing to the uncertainty (used in 71 studies) [46].

## Discussion

### Methodological considerations

A limitation of mapping reviews is that they tend to be time constrained [29]. Furthermore, mapping reviews do not usually include any quality assessment of the included studies [29]. On the other hand, mapping reviews serves as an excellent starting point for initiating new research and reviews. The major contribution of this review is that it provides an overview of the body of research addressing the key concepts, across different fields of research. We have used pragmatic, but explicit criteria guided by a list of the key concepts we believe is important for people to be able to understand and assess treatment claims. Our overview provides information about which key concepts have been targets of interventions, how understanding and skills have been evaluated, and which concepts have received little attention in research. As noted in the introduction, the assessment of people’s assessments of treatment effects is challenging both because no inventory of key concepts underpinning such understanding and skills has been available until recently, and by parallel areas of research that are only partly overlapping. The findings of this review provide a starting point for decisions about which concepts to address when developing new interventions, and which additional assessment tools should be considered.

This review did not attempt to compare the effects of such interventions, this should be done by using other review methods. Related to this review is an ongoing review on the effects of educational interventions to improve people’s understanding of the key concepts [47].

Applying the key concept list to the identified research literature, we sorted the interventions into four categories: risk and DA, EBM, and CA, understanding of controlled trials, and science education. Overall, interventions focused on a small number of key concepts, and typically targeted the same concepts within each research area. The most common key concepts in interventions were concepts 5.1 “Treatments usually have beneficial and harmful effects,” concepts 2.1 “Treatment comparisons should be fair,” 2.2 “Compare like with like” (117 studies) and concept 4.1 “Single studies can be misleading”. A variety of assessment tools were identified, with approximately 210 discreet tools and procedures. Four assessment tools included 10 or more key concepts, but none of these instruments targeted patients or consumers. The most frequently used assessment tools were the Fresno tool and the Decisional Conflict Scale [44, 46].

A challenge we had conducting this review was that the descriptions and reporting of interventions and assessment tools were often limited. In cases in which key concepts were not explicitly stated as part of the intervention or assessment tools, we did not attempt to make any assumptions about whether concepts could have been included. A typical example would be decision aids, for which key concepts such as risk are usually presented as part of the decision aid and concept 5.2 “relative effects can be misleading” is likely to be relevant. In such cases, we did not assume that this concept was considered unless educating people about this concept was an explicitly reported component of the intervention. Another example is studies evaluating the effects of interventions teaching people EBM and CA skills. It was often unclear and rarely reported which concepts were included in these interventions and assessment tools. As a result, we may have missed relevant interventions or assessment tools that addressed some of the key concepts, or the number of concepts included may be underreported. Furthermore, the understanding of certain key concepts has changed over the years, for example, the reporting of *p* values and confidence intervals (concepts 3.2 and 3.3). In cases where the interpretation of these concepts was not explicit, we did not include them.

Another limitation of our review is that our search for studies was deliberately focused, and may not have identified all studies or assessment tools targeting one or more of our key concepts. However, we are quite certain, based on the large number of studies we included, that we have probably identified the most relevant interventions and assessment tools targeting the key concepts in our list. The list of key concepts is an evolving document, which will undergo yearly revisions where new key concepts may be added or existing concepts may be revised. While conducting this review, one concept was added and one concept was divided into two concepts. This did not have any implication for the review methods or results, other than that we did not map or identify instruments that addressed this newly added concept.

We included interventions and assessment tools used for both patients and health professionals. Patient education in critical thinking is fairly new, and we believe that when developing such interventions researchers and others may learn from what has been done in interventions developed for health professionals. Evidence also suggests that patients and health professionals have many of the same needs when it comes to training in the key concepts. For example, in studies evaluating specific concepts such as risk, no differences were found between patients and health professionals in understanding of different statistical formats of risk [27]. Furthermore, evidence-based practice and the need for fair tests of treatments has yet to be universally acknowledged [48, 49]. Thus, many professionals may not have had training in these concepts as part of their professional training. Therefore, interventions and assessment tools relevant for professionals may also be relevant to patients and vice versa, although terminology and examples used in such training may differ [50].

We did not assess the quality of the assessment tools we identified using COSMIN or other checklists [51]. This was because the main purpose of this review was to identify an assessment tool we could use to evaluate interventions targeted at the concepts in the list. If we had identified such instruments, the next step in this process would have been to ascertain the quality of these. However, none of the studies we found included more than half of the key concepts.

Nearly half of the excluded studies were “health literacy” studies. Health literacy has been defined in many ways but generally encompasses people’s ability to find, assess and apply reliable health information [26]. The most commonly used instruments in this area concentrate on measuring functional literacy, that is, general reading or numeracy skills, such as the Test for Functional Health Literacy in Adults (TOFHLA) or the Rapid Estimate of Adult Literacy (REALM) [52, 53]. Some instruments also include critical appraisal skills; however, none of the health literacy instruments we found addressed the key concepts directly. Instead, they measured more general understanding of health information and medical terminology [54–56].

Many school systems perform national assessments of school children’s science and mathematical literacy to measure educational achievement, such as the PISA test or SAT’s [57, 58]. We identified several such assessment tools; however, none of these met our inclusion criteria. This may be because many of these instruments are not publicly available and are apparently subject to change on a regular basis. Furthermore, such instruments generally focus on measuring understanding of basic science and mathematics. Although we found that many instruments included content relevant to our concepts, such as the importance of supporting claims by research evidence, preparing a protocol, conducting laboratory experiments and calculating probabilities, they did not address our key concepts directly.

## Conclusions

The findings of this review indicate that the key concepts people need to understand to assess claims about treatment effects are of interdisciplinary research interest. However, the interventions we identified, and assessment tools used to map or evaluate peoples’ understanding, included only a handful of the key concepts. This suggests that many of the key concepts have not been focus of research, and that there is great need to explore how understanding about these key concepts can be improved and how such understanding can be evaluated. The findings of this review consequently should inform future research priorities, such as the choice of key concepts to include in interventions and for considering appropriate outcomes and assessment tools.

## Additional files

Additional file 1: Short list of Key Concepts. (DOCX 14 kb)
Additional file 2: Search strategy. (DOCX 30 kb)
Additional file 3: Overview of all included studies. (XLSX 133 kb)

## Abbreviations

Key concepts: Key concepts that people need to understand to assess treatment claims
REALM: Rapid estimate of adult literacy
TOFHLA: Test for functional health literacy in adults
