# Supplementary material for: Interventions and assessment tools addressing key concepts people need to know to appraise claims about treatment effects: a systematic mapping review
Source: Syst Rev. 2016 Dec 29;5:215. doi: 10.1186/s13643-016-0389-z (PMC5200965; doi:10.1186/s13643-016-0389-z)
Supplement: Additional file 1: — Short list of Key Concepts. (DOCX 14 kb) [file 13643_2016_389_MOESM1_ESM.docx]

| **Informed Healthcare Choices Concepts** |
| --- |
| 1. Recognising the need for fair comparisons of treatments  [Fair treatment comparisons are needed] |
| 1.1 Treatments may be harmful [Treatments can harm] |
| 1.2 Personal experiences or anecdotes (stories) are an unreliable basis for determining the effects of most treatments [Anecdotes are not reliable evidence] |
| 1.3 A treatment [outcome](#outcome) may be [associated](#association) with a treatment, but not caused by the treatment [Association is not necessarily causation] |
| 1.4 Widely used or traditional treatments are not necessarily beneficial or safe [Practice is often not based on evidence] |
| 1.5 New, brand-named, or more expensive treatments may not be better than available alternatives [New treatments are not always better] |
| 1.6 Opinions of experts or authorities do not alone provide a reliable basis for deciding on the benefits and harms of treatments [Expert opinion is not always right] |
| 1.7 Conflicting interests may result in misleading claims about the effects of treatments [Be aware of conflicts of interest] |
| 1.8 Increasing the amount of a treatment does not necessarily increase the benefits of a treatment and may cause harm [More is not necessarily better] |
| 1.9 Earlier detection of disease is not necessarily better [Earlier is not necessarily better] |
| 1.10 Hope can lead to unrealistic expectations about the effects of treatments [Avoid unrealistic expectations] |
| 1.11 Beliefs about how treatments work are not reliable predictors of the actual effects of treatments [Theories about treatment can be wrong] |
| 1.12 Large, dramatic effects of treatments are rare [Dramatic treatment effects are rare] |
|  |
| 2. Judging whether a comparison of treatments is a fair comparison  [Treatment comparisons should be fair] |
| 2.1 Evaluating the effects of treatments requires appropriate comparisons [Treatment comparisons are necessary] |
| 2.2 Apart from the treatments being compared, the comparison groups need to be similar (i.e. 'like needs to be compared with like') [Compare like with like] |
| 2.3 People’s experiences should be counted in the group to which they were allocated  [Base analyses on allocated treatment] |
| 2.4 People in the groups being compared need to be cared for similarly (apart from the treatments being compared)  [Treat comparison groups similarly] |
| 2.5 If possible, people should not know which of the treatments being compared they are receiving [Blind participants to their treatments] |
| 2.6 Outcomes should be measured in the same way (fairly) in the treatment groups being compared  [Assess outcome measures fairly] |
| 2.7 It is important to measure outcomes in everyone who was included in the treatment comparison groups  [Follow up everyone included] |
|  |
| 3. Understanding the role of [chance](#chance) [Understand the role of chance] |
| 3.1 Small studies in which few outcome events occur are usually not informative and the results may be misleading  [Small studies may be misleading] |
| 3.2 The use of [p-values](#p) to indicate the [probability](#probability) of something having occurred by chance may be misleading; [confidence intervals](#confidence) are more informative [P-values alone can be misleading] |
| 3.3 Saying that a difference is [statistically significant](#stastical) or that it is not statistically significant can be misleading [‘Significance’ may be misleading] |
|  |
| 4. Considering all of the relevant fair comparisons  [Consider all the relevant evidence] |
| 4.1 The results of single tests of treatments can be misleading  [Single studies can be misleading] |
| 4.2 Reviews of treatment tests that do not use systematic methods can be misleading  [Unsystematic reviews can mislead] |
| 4.3 Well done systematic reviews often reveal a lack of relevant evidence, but they provide the best basis for making judgements about the certainty of the evidence  [Consider how certain the evidence is] |
|  |
| 5. Understanding the results of fair comparisons of treatments  [Understand the results of comparisons] |
| 5.1 Treatments may have beneficial and harmful effects  [Weigh benefits and harms of treatment] |
| 5.2 [Relative effects](#relative) of treatments alone can be misleading  [Relative effects can be misleading] |
| 5.3 [Average differences](#average) between treatments can be misleading  [Average differences can be misleading] |
|  |
| 6. Judging whether fair comparisons of treatments are relevant  [Judge relevance of fair comparisons] |
| 6.1 Fair comparisons of treatments should measure outcomes that are important  [Outcomes studied may not be relevant] |
| 6.2 Fair comparisons of treatments in animals or highly selected groups of people may not be relevant  [People studied may not be relevant] |
| 6.3 The treatments evaluated in fair comparisons may not be relevant or applicable  [Treatments used may not be relevant] |
| 6.4 Results for a selected group of people within fair comparisons can be misleading  [Beware of subgroup analyses] |

Table 1. Short list of key concepts people need to understand to assess claims about treatment effects
