# Supplementary material for: Interventions and assessment tools addressing key concepts people need to know to appraise claims about treatment effects: a systematic mapping review
Source: Syst Rev. 2016 Dec 29;5:215. doi: 10.1186/s13643-016-0389-z (PMC5200965; doi:10.1186/s13643-016-0389-z)
Supplement: Additional file 2: — Search strategy. (DOCX 30 kb) [file 13643_2016_389_MOESM2_ESM.docx]

**Search strategies**

| **Database name**  **Database time span**  **Database host** | **Date searched** |
| --- | --- |
| CDSR (Cochrane Library) | 21.-22.06.13 |
| DARE (Cochrane Library) |  |
| HTA (Cochrane Library) |  |
| CENTRAL (Cochrane Library) |  |
| Method studies (Cochrane Library) | 21.06.13 |
| MEDLINE In-Process & Other Non-Indexed Citations, and MEDLINE 1946 to Present (Ovid) | 22.06.13 |
| ERIC 1966 to present (ProQuest) | 21.06.13 |

**CDSR, DARE, HTA, CENTRAL (Cochrane Library)**

| #1 | MeSH descriptor: [Information Literacy] this term only | 3 |
| --- | --- | --- |
| #2 | MeSH descriptor: [Health Literacy] this term only | 61 |
| #3 | ("information literacy" or "numerical literacy" or "statistical literacy" or "research literacy" or "science literacy" or "scientific literacy") | 14 |
| #4 | understand next (effect or risk or probability or causality or randomisation or randomization) | 46 |
| #5 | understand near/3 ("control group" or "control groups" or "comparison group" or "comparison groups" or "compare groups") | 9 |
| #6 | (critical* next apprais* or critical* next read* or "evidence based") near/3 (knowledge or skill or skills or competen*) | 178 |
| #7 | (health* next information or "health care information" or "medical information" or health* next advice or "health care advice" or "medical advice" or health* next research or "health care research" or "medical research" or numeracy or number or numbers or health* next statistics or "health care statistics" or "medical statistics" or health* next science or "health care science" or "medical science" or "scientific information" or "controlled trial" or "controlled trials" or "controlled clinical trial" or "controlled clinical trials" or controlled next stud* or controlled next clinical next stud* or RCT or RCTs or systematic next review? or "evidence based") near/3 (understand* or comprehend or comprehension or literacy or critical* next apprais*) | 197 |
| #8 | "testing treatments" | 13 |
| #9 | #1 or #2 or #3 or #4 or #5 or #6 or #7 or #8 | 494 |
| #10 | MeSH descriptor: [Clinical Trials as Topic] explode all trees | 53646 |
| #11 | MeSH descriptor: [Evidence-Based Practice] explode all trees | 1842 |
| #12 | #10 or #11 | 54945 |
| #13 | MeSH descriptor: [Comprehension] this term only | 289 |
| #14 | #12 and #13 | 29 |
| #15 | #9 or #14  in Cochrane Reviews (Reviews and Protocols), Other Reviews, Trials and Technology Assessments | 397 |

**Methods studies (Cochrane Library)**

| #1 | "critical appraisal" and (general or internet):kw | 590 |
| --- | --- | --- |
| #2 | (knowledge or skill or skills or competen*) | 25343 |
| #3 | #1 and #2 | 51 |
| #4 | ("information literacy" or "numerical literacy" or "statistical literacy" or "research literacy" or "science literacy" or "scientific literacy") | 14 |
| #5 | understand next (effect or risk or probability or causality or randomisation or randomization) | 46 |
| #6 | understand near/3 ("control group" or "control groups" or "comparison group" or "comparison groups" or "compare groups") | 9 |
| #7 | (critical* next apprais* or critical* next read* or "evidence based") near/3 (knowledge or skill or skills or competen*) | 178 |
| #8 | (health* next information or "health care information" or "medical information" or health* next advice or "health care advice" or "medical advice" or health* next research or "health care research" or "medical research" or numeracy or number or numbers or health* next statistics or "health care statistics" or "medical statistics" or health* next science or "health care science" or "medical science" or "scientific information" or "controlled trial" or "controlled trials" or "controlled clinical trial" or "controlled clinical trials" or controlled next stud* or controlled next clinical next stud* or RCT or RCTs or systematic next review? or "evidence based") near/3 (understand* or comprehend or comprehension or literacy or critical* next apprais*) | 197 |
| #9 | "testing treatments" | 13 |
| #10 | #3 or #4 or #5 or #6 or #7 or #8 or #9  in Methods Studies | 149 |

**MEDLINE (Ovid)**

| **#** | **Searches** | **Results** |
| --- | --- | --- |
| 1 | Information Literacy/ | 77 |
| 2 | Health Literacy/ | 1327 |
| 3 | information literacy.ti,ab. | 203 |
| 4 | (numerical literacy or statistical literacy or research literacy or science literacy or scientific literacy).ti,ab. | 153 |
| 5 | (understand* adj effect).ti,ab. | 13 |
| 6 | (understand* adj risk).ti,ab. | 297 |
| 7 | (understand* adj probability).ti,ab. | 5 |
| 8 | (understand* adj causality).ti,ab. | 21 |
| 9 | (understand* adj (randomisation or randomization)).ti,ab. | 7 |
| 10 | (understand* adj3 (control group? or comparison group?)).ti,ab. | 15 |
| 11 | ((critical* apprais* or critical* read* or evidence based) adj3 (knowledge or skill? or competen*)).ti,ab. | 1276 |
| 12 | ((health* information or health care information or medical information or health* advice or health care advice or medical advice or health* research or health care research or medical research or numeracy or number? or health* statistics or health care statistics or medical statistics or health* science or health care science or medical science or scientific information or controlled trial? or controlled clinical trial? or controlled stud* or controlled clinical stud* or RCT? or systematic review? or evidence based) adj3 (understand* or comprehend or comprehension or literacy or critical* apprais*)).ti,ab. | 1515 |
| 13 | testing treatments.ti,ab. | 54 |
| 14 | or/1-13 | 4660 |
| 15 | exp Clinical Trials as Topic/ | 277944 |
| 16 | exp Evidence-Based Practice/ | 58457 |
| 17 | 15 or 16 | 327017 |
| 18 | Comprehension/ | 7541 |
| 19 | 17 and 18 | 391 |
| 20 | 14 or 19 | 5020 |
| 21 | limit 20 to "reviews (maximizes specificity)" | 394 |
| 22 | randomized controlled trial.pt. | 367706 |
| 23 | controlled clinical trial.pt. | 87723 |
| 24 | (randomis* or randomiz* or randomly).ti,ab. | 535075 |
| 25 | groups.ti,ab. | 1321153 |
| 26 | trial.ti. | 121765 |
| 27 | or/22-26 [Based on the Cochrane HSSS] | 1843678 |
| 28 | 20 and 27 | 1070 |
| 29 | Epidemiologic studies/ | 5920 |
| 30 | exp case control studies/ | 628445 |
| 31 | exp cohort studies/ | 1303689 |
| 32 | case control.tw. | 73718 |
| 33 | (cohort adj (study or studies)).tw. | 79971 |
| 34 | cohort analy*.tw. | 3443 |
| 35 | (follow up adj (study or studies)).tw. | 36536 |
| 36 | (observational adj (study or studies)).tw. | 41109 |
| 37 | longitudinal.tw. | 134443 |
| 38 | retrospective.tw. | 257329 |
| 39 | cross sectional.tw. | 157879 |
| 40 | Cross-sectional studies/ | 166258 |
| 41 | or/29-40 [Observational filter from SIGN] | 1810712 |
| 42 | 20 and 41 | 696 |
| 43 | 21 or 28 or 42 | 1810 |

**ERIC (ProQuest)**

| S1 | ALL("information literacy") or ALL("health literacy") or ALL("numerical literacy") or ALL("statistical literacy") or ALL("research literacy") or ALL(science lieteracy) or ALL(scientific literacy) | 7094* |
| --- | --- | --- |
| S2 | ALL(understand* pre/0 effect) or ALL(understand* pre/0 risk) or ALL(understand* pre/0 probability) or ALL(understand* pre/0 causality) or ALL(understand* pre/0 randomisation) or ALL(understand* pre/0 randomization) or ALL(understand* pre/3 "control group") or ALL(understand* pre/3 "control groups") or ALL(understand* pre/3 "comparison group") or ALL(understand* pre/3 "comparison groups") | 65° |
| S3 | ALL(critical* pre/0 apprais*) or ALL(critical* pre/0 read*) or ALL("evidence based") | 6748* |
| S4 | ALL(knowledge or skill or skills or competen*) | 339280* |
| S5 | S3 AND S4 | 2716° |
| S6 | ALL(understand* pre/3 "health information") or ALL(understand* pre/3 "healthcare information") or ALL(understand* pre/3 "health care information") or ALL(understand* pre/3 "medical information") or ALL(understand* pre/3 "health advice") or ALL(understand* pre/3 "healthcare advice") or ALL(understand* pre/3 "health care advice") or ALL(understand* pre/3 "medical advice") or ALL(understand* pre/3 "health research") or ALL(understand* pre/3 "healthcare research") or ALL(understand* pre/3 "health care research") or ALL(understand* pre/3 "medical research") or ALL(understand* pre/3 numeracy) or ALL(understand* pre/3 number) or ALL(understand* pre/3 numbers) or ALL(understand* pre/3 "health statistics") or ALL(understand* pre/3 "healthcare statistics") or ALL(understand* pre/3 "health care statistics") or ALL(understand* pre/3 "medical statistics") or ALL(understand* pre/3 "health science") or ALL(understand* pre/3 "healthcare science") or ALL(understand* pre/3 "health care science") or ALL(understand* pre/3 "medical science") or ALL(understand* pre/3 "scientific information") or ALL(understand* pre/3 "controlled trial") or ALL(understand* pre/3 "controlled trials") or ALL(understand* pre/3 "controlled clinical trial") or ALL(understand* pre/3 "controlled clinical trials") or ALL(understand* pre/3 "controlled study") or ALL(understand* pre/3 "controlled studies") or ALL(understand* pre/3 "controlled clinical study") or ALL(understand* pre/3 "controlled clinical studies") or ALL(understand* pre/3 RCT) or ALL(understand* pre/3 RCTs) or ALL(understand* pre/3 "systematic review") or ALL(understand* pre/3 "systematic reviews") or ALL(understand* pre/3 "evidence based") | 403° |
| S7 | ALL(comprehend pre/3 "health information") or ALL(comprehend pre/3 "healthcare information") or ALL(comprehend pre/3 "health care information") or ALL(comprehend pre/3 "medical information") or ALL(comprehend pre/3 "health advice") or ALL(comprehend pre/3 "healthcare advice") or ALL(comprehend pre/3 "health care advice") or ALL(comprehend pre/3 "medical advice") or ALL(comprehend pre/3 "health research") or ALL(comprehend pre/3 "healthcare research") or ALL(comprehend pre/3 "health care research") or ALL(comprehend pre/3 "medical research") or ALL(comprehend pre/3 numeracy) or ALL(comprehend pre/3 number) or ALL(comprehend pre/3 numbers) or ALL(comprehend pre/3 "health statistics") or ALL(comprehend pre/3 "healthcare statistics") or ALL(comprehend pre/3 "health care statistics") or ALL(comprehend pre/3 "medical statistics") or ALL(comprehend pre/3 "health science") or ALL(comprehend pre/3 "healthcare science") or ALL(comprehend pre/3 "health care science") or ALL(comprehend pre/3 "medical science") or ALL(comprehend pre/3 "scientific information") or ALL(comprehend pre/3 "controlled trial") or ALL(comprehend pre/3 "controlled trials") or ALL(comprehend pre/3 "controlled clinical trial") or ALL(comprehend pre/3 "controlled clinical trials") or ALL(comprehend pre/3 "controlled study") or ALL(comprehend pre/3 "controlled studies") or ALL(comprehend pre/3 "controlled clinical study") or ALL(comprehend pre/3 "controlled clinical studies") or ALL(comprehend pre/3 RCT) or ALL(comprehend pre/3 RCTs) or ALL(comprehend pre/3 "systematic review") or ALL(comprehend pre/3 "systematic reviews") or ALL(comprehend pre/3 "evidence based") | 2° |
| S8 | ALL(comprehension pre/3 "health information") or ALL(comprehension pre/3 "healthcare information") or ALL(comprehension pre/3 "health care information") or ALL(comprehension pre/3 "medical information") or ALL(comprehension pre/3 "health advice") or ALL(comprehension pre/3 "healthcare advice") or ALL(comprehension pre/3 "health care advice") or ALL(comprehension pre/3 "medical advice") or ALL(comprehension pre/3 "health research") or ALL(comprehension pre/3 "healthcare research") or ALL(comprehension pre/3 "health care research") or ALL(comprehension pre/3 "medical research") or ALL(comprehension pre/3 numeracy) or ALL(comprehension pre/3 number) or ALL(comprehension pre/3 numbers) or ALL(comprehension pre/3 "health statistics") or ALL(comprehension pre/3 "healthcare statistics") or ALL(comprehension pre/3 "health care statistics") or ALL(comprehension pre/3 "medical statistics") or ALL(comprehension pre/3 "health science") or ALL(comprehension pre/3 "healthcare science") or ALL(comprehension pre/3 "health care science") or ALL(comprehension pre/3 "medical science") or ALL(comprehension pre/3 "scientific information") or ALL(comprehension pre/3 "controlled trial") or ALL(comprehension pre/3 "controlled trials") or ALL(comprehension pre/3 "controlled clinical trial") or ALL(comprehension pre/3 "controlled clinical trials") or ALL(comprehension pre/3 "controlled study") or ALL(comprehension pre/3 "controlled studies") or ALL(comprehension pre/3 "controlled clinical study") or ALL(comprehension pre/3 "controlled clinical studies") or ALL(comprehension pre/3 RCT) or ALL(comprehension pre/3 RCTs) or ALL(comprehension pre/3 "systematic review") or ALL(comprehension pre/3 "systematic reviews") or ALL(comprehension pre/3 "evidence based") | 111° |
| S9 | ALL("critical appraisal" pre/3 "health information") or ALL("critical appraisal" pre/3 "healthcare information") or ALL("critical appraisal" pre/3 "health care information") or ALL("critical appraisal" pre/3 "medical information") or ALL("critical appraisal" pre/3 "health advice") or ALL("critical appraisal" pre/3 "healthcare advice") or ALL("critical appraisal" pre/3 "health care advice") or ALL("critical appraisal" pre/3 "medical advice") or ALL("critical appraisal" pre/3 "health research") or ALL("critical appraisal" pre/3 "healthcare research") or ALL("critical appraisal" pre/3 "health care research") or ALL("critical appraisal" pre/3 "medical research") or ALL("critical appraisal" pre/3 numeracy) or ALL("critical appraisal" pre/3 number) or ALL("critical appraisal" pre/3 numbers) or ALL("critical appraisal" pre/3 "health statistics") or ALL("critical appraisal" pre/3 "healthcare statistics") or ALL("critical appraisal" pre/3 "health care statistics") or ALL("critical appraisal" pre/3 "medical statistics") or ALL("critical appraisal" pre/3 "health science") or ALL("critical appraisal" pre/3 "healthcare science") or ALL("critical appraisal" pre/3 "health care science") or ALL("critical appraisal" pre/3 "medical science") or ALL("critical appraisal" pre/3 "scientific information") or ALL("critical appraisal" pre/3 "controlled trial") or ALL("critical appraisal" pre/3 "controlled trials") or ALL("critical appraisal" pre/3 "controlled clinical trial") or ALL("critical appraisal" pre/3 "controlled clinical trials") or ALL("critical appraisal" pre/3 "controlled study") or ALL("critical appraisal" pre/3 "controlled studies") or ALL("critical appraisal" pre/3 "controlled clinical study") or ALL("critical appraisal" pre/3 "controlled clinical studies") or ALL("critical appraisal" pre/3 RCT) or ALL("critical appraisal" pre/3 RCTs) or ALL("critical appraisal" pre/3 "systematic review") or ALL("critical appraisal" pre/3 "systematic reviews") or ALL("critical appraisal" pre/3 "evidence based") | 1° |
| S10 | ALL("critically appraise" pre/3 "health information") or ALL("critically appraise" pre/3 "healthcare information") or ALL("critically appraise" pre/3 "health care information") or ALL("critically appraise" pre/3 "medical information") or ALL("critically appraise" pre/3 "health advice") or ALL("critically appraise" pre/3 "healthcare advice") or ALL("critically appraise" pre/3 "health care advice") or ALL("critically appraise" pre/3 "medical advice") or ALL("critically appraise" pre/3 "health research") or ALL("critically appraise" pre/3 "healthcare research") or ALL("critically appraise" pre/3 "health care research") or ALL("critically appraise" pre/3 "medical research") or ALL("critically appraise" pre/3 numeracy) or ALL("critically appraise" pre/3 number) or ALL("critically appraise" pre/3 numbers) or ALL("critically appraise" pre/3 "health statistics") or ALL("critically appraise" pre/3 "healthcare statistics") or ALL("critically appraise" pre/3 "health care statistics") or ALL("critically appraise" pre/3 "medical statistics") or ALL("critically appraise" pre/3 "health science") or ALL("critically appraise" pre/3 "healthcare science") or ALL("critically appraise" pre/3 "health care science") or ALL("critically appraise" pre/3 "medical science") or ALL("critically appraise" pre/3 "scientific information") or ALL("critically appraise" pre/3 "controlled trial") or ALL("critically appraise" pre/3 "controlled trials") or ALL("critically appraise" pre/3 "controlled clinical trial") or ALL("critically appraise" pre/3 "controlled clinical trials") or ALL("critically appraise" pre/3 "controlled study") or ALL("critically appraise" pre/3 "controlled studies") or ALL("critically appraise" pre/3 "controlled clinical study") or ALL("critically appraise" pre/3 "controlled clinical studies") or ALL("critically appraise" pre/3 RCT) or ALL("critically appraise" pre/3 RCTs) or ALL("critically appraise" pre/3 "systematic review") or ALL("critically appraise" pre/3 "systematic reviews") or ALL("critically appraise" pre/3 "evidence based") | 1° |
| S11 | ALL("testing treatments") | 1° |
| S12 | S1 OR S2 OR S5 OR S6 OR S7 OR S8 OR S9 OR S10 OR S11 | 10309* |
| S13 | ALL(medic* or health* or treatment*) | 168249* |
| S14 | S12 AND S13 | 1263° |
| S15 | ALL(review or "controlled trial" or "controlled study" or randomis* or randomiz* or randomly or epidemiologic or "case control" or "cohort study" or "cohort analysis" or "follow up study" or observational or longitudinal or retrospective or "cross sectional") | 218642* |
| S16 | S14 AND S15 | 283° |
